# Oral Delivery of Pentameric Glucagon-Like Peptide-1 by Recombinant *Lactobacillus* in Diabetic Rats

**DOI:** 10.1371/journal.pone.0162733

**Published:** 2016-09-09

**Authors:** Yin Lin, Kasper Krogh-Andersen, Julien Pelletier, Harold Marcotte, Claes-Göran Östenson, Lennart Hammarström

**Affiliations:** 1 Department of Laboratory Medicine, Division of Clinical Immunology and Transfusion Medicine, Karolinska Institutet at Karolinska University Hospital Huddinge, SE-141 86 Stockholm, Sweden; 2 Department of Molecular Medicine and Surgery, Rolf Luft Research Center for Diabetes and Endocrinology, M1:03 Karolinska University Hospital, Karolinska Institutet, Stockholm SE-17176, Sweden; Johns Hopkins School of Medicine, UNITED STATES

## Abstract

Glucagon-like peptide-1 (GLP-1) is an incretin hormone produced by intestinal cells and stimulates insulin secretion from the pancreas in a glucose-dependent manner. Exogenously supplied GLP-1 analogues are used in the treatment of type 2 diabetes. An anti-diabetic effect of *Lactobacillus* in lowering plasma glucose levels and its use as a vehicle for delivery of protein and antibody fragments has been shown previously. The aim of this study was to employ lactobacilli as a vehicle for *in situ* production and delivery of GLP-1 analogue to normalize blood glucose level in diabetic GK (Goto-Kakizaki) rats. In this study, we designed pentameric GLP-1 (5×GLP-1) analogues which were both expressed in a secreted form and anchored to the surface of lactobacilli. Intestinal trypsin sites were introduced within 5×GLP-1, leading to digestion of the pentamer into an active monomeric form. The *E*. *coli*-produced 5×GLP-1 peptides delivered by intestinal intubation to GK rats resulted in a significant improvement of glycemic control demonstrated by an intraperitoneal glucose tolerance test. Meanwhile, the purified 5×GLP-1 (trypsin-digested) from the *Lactobacillus* cultures stimulated insulin secretion from HIT-T15 cells, similar to the *E*. *coli*-produced 5×GLP-1 peptides. When delivered by gavage to GK rats, non-expressor *L*. *paracasei* significantly lowered the blood glucose level but 5×GLP-1 expression did not provide an additional anti-diabetic effect, possibly due to the low levels produced. Our results indicate that lactobacilli themselves might be used as an alternative treatment method for type 2 diabetes, but further work is needed to increase the expression level of GLP-1 by lactobacilli in order to obtain a significant insulinotropic effect *in vivo*.

## Introduction

Type 2 diabetes is a metabolic disorder characterized by high blood glucose due to insulin resistance and relative insulin deficiency [1]. Glucagon-like peptide-1 (GLP-1), which is a proglucagon-derived peptide produced by intestinal L cells, is used for treatment of type 2 diabetes. GLP-1 is an incretin hormone that is secreted in response to nutrient ingestion. It stimulates insulin secretion from the pancreas in a glucose-dependent manner, suppresses glucagon secretion and slows down gastric emptying [2]. GLP-1 can also reduce food intake and body weight in obese patients with type 2 diabetes [3, 4]. Many studies have demonstrated the promising potential of exogenously supplied GLP-1 in the treatment of type 2 diabetes as it can normalize glucose levels following subcutaneous injections in diabetic patients [5, 6]. Furthermore, GLP-1-induced stimulation of insulin secretion is strictly glucose-dependent and does not cause hypoglycemia, a severe side effect of some medications presently used for the treatment of diabetes [7].

The amino acid sequence of GLP-1 is highly conserved in mammals [8]. The inactive full-length form of GLP-1 (1–37) is processed into two active circulating forms, GLP-1 (7–37) and GLP-1 (7–36) amide, with the latter being the most abundant form found in blood. Both forms of GLP-1 are sensitive to dipeptidyl peptidase-IV (DPP-IV) digestion in serum, and the native form of GLP-1 has a very short half-life (less than 2 minutes) where concentrations return to baseline within 90 min after subcutaneous injection [9], making it difficult to administer systemically. Current research has focused on developing long-acting GLP-1 receptor agonists such as Exenatide (Byetta^®^), a GLP-1 analogue originally found in the saliva of the Gila monster. It has a 53% amino acid identity to GLP-1 and a decreased sensitivity to DPP-IV, resulting in a longer half-life (2.4 h) *in vivo* [10]. Liraglutide (Victoza^®^) is a GLP-1 analogue that shares 97% sequence identity with GLP-1. The addition of a C16 fatty acid side chain facilitates binding of the drug to circulating serum albumin, prolonging its duration of action to 24 h and enabling once-daily injection of the peptide [11]. Furthermore, the replacement of alanine by glycine in position 8 (GLP-1-Gly8), as also utilized in Exenatide, significantly increases the insulinotropic effect through improved resistance against proteolytic inactivation by DPP-IV [12].

Since GLP-1 is secreted from the distal ileum and colon, it is found in highest concentration in the splanchnic blood and is not equally distributed throughout the systemic circulation [13]. Therefore, the current therapeutic route (subcutaneous injection), does not strictly mimic the physiological release of GLP-1 [13, 14]. In contrast, oral delivery of peptides followed by uptake through the intestine would more likely mimic physiological GLP-1 secretion while providing a more convenient and comfortable drug delivery method for patients. Substantial efforts have previously been made to overcome the oral delivery problem by adding novel functional groups to facilitate absorption [15], by PEGylation or encapsulating GLP-1 into nanoparticles to protect the peptides from degradation by proteases in the gastrointestinal tract [16–18].

Lactobacilli are Gram-positive bacteria that have been historically used in food fermentation and preservation. They are also normal residents of the gastrointestinal tract of animals and humans and formally recognized as “generally recognized as safe” (GRAS) organisms [19]. Some *Lactobacillus* strains can survive the gastrointestinal passage and colonize the gastrointestinal tract where they can be utilized for direct *in situ* delivery of peptides or proteins, reducing their exposure to gastric acid, bile and digestive enzymes. This would provide a continuous supply of biologically active peptides, which, after absorption through epithelial cells, could interact with receptors. Some strains of *Lactobacillus* have previously been shown to exert an anti-diabetic effect in animal models [20, 21], possibly increasing the effect if used as a vehicle for delivery of GLP-1. In addition, a recent publication demonstrated that feeding with a *Lactobacillus gasseri* delivering “receptor-inactive” full-length GLP-1 (1–37) for a 90 day period, could reprogram intestinal cells into glucose-responsive insulin-secreting cells in a type-1-diabetic rat model [22], suggesting that lactobacilli are a potent candidate for active GLP-1 (7–37) peptide delivery.

Antibody fragments have previously been expressed by lactobacilli to combat viral and bacterial infections in the gastrointestinal tract [23, 24]. In this study, we engineered a *Lactobacillus* strain to express a pentameric form of GLP-1 peptide containing five tandem repeated GLP-1 analogs (GLP-1-Gly8) in both secreted and cell wall-anchored forms. The pentameric GLP-1 was digested by the intestinal trypsin and monomeric GLP-1 was released in the gut. The bioactivity of this GLP-1 analog was subsequently tested in an *in vitro* model and in a diabetic rat model.

## Materials and Methods

### Bacterial strains, plasmids and culture conditions

*E*. *coli* DH5α (Invitrogen, Carlsbad, CA) was grown in Luria-Bertani (LB) broth at 37°C with 200 rpm orbital shaking or on LB-agar plates at 37°C. *Lactobacillus paracasei* BL23 (previously named *L*. *casei* 393 pLZ15-) [25, 26] was inoculated in MRS broth (Difco, Sparks, MD) at OD_600_ = 0.08 from overnight culture and grown at 37°C without agitation to an OD_600_ equal to 1.0 (2×10^8^ cfu/ml) or anaerobically (BD—GasPak EZ, Sparks, MD) on MRS-agar plates. When required, erythromycin was added as follows: 300 μg/ml for *E*. *coli* DH5α and 5 μg/ml for *L*. *paracasei* BL23.

### Synthesis of peptides

The GLP-1 peptide and its analogue were produced synthetically by GenScript Corporation (Piscataway, NJ) based on the bioactive GLP-1(7–37) form: (1) GLP-1 (98.3% purity), a 31 amino acid (aa) peptide corresponding to the conserved sequence found in all mammals; (2) GLP-1-Gly8 (97.5% purity), a 31 aa peptide of GLP-1 with the alanine (Ala) in position 2 changed into glycine (Gly), protecting the peptide against enzymatic digestion by DPP-IV by altering the cleavage site for the peptidase [12].

### Construction of recombinant *Lactobacillus* expressing pentameric GLP-1

The wild type (wt) 5xGLP-1 and trypsin stabilized (trp) 5xGLP-1 genes were produced as synthetic genes and codons optimized for expression in *L*. *paracasei* (GenScript, Piscataway, NJ) (S1 Table). The synthetic genes, flanked by an upstream *Nco*I and a downstream *Not*I restriction site, were excised using these two restriction enzymes (Thermo Scientific, Waltham, MA) and ligated into the *Nco*I/*Not*I digested *Lactobacillus* expression vectors pAF100 and pAF900 for secreted expression and cell wall anchored expression respectively [27]. Four expression plasmids were constructed for production of the wt 5xGLP-1-secreted (pKA488) and cell wall-anchored (pKA489) as well as the trp 5xGLP-1-secreted (pKA480) and cell wall-anchored (pKA486) genes. The expression plasmids were transformed into *E*. *coli* DH5α competent cells by electroporation and the DNA sequence of the expression cassettes were verified. The plasmids were subsequently transformed into *L*. *paracasei* BL23 [26] by electroporation as previously described [28, 29], generating *Lactobacillus* strains expressing 5×GLP-1 which were denoted Lp wt 5×GLP-1 or Lp trp 5×GLP-1 (Table 1).

**Table 1:** Strains and plasmids used in this study.

Plasmids	*L*. *paracasei* strain	Products in *L*. *paracasei*	Reference
pKA480	KKA394	Secreted trypsin stabilized 5×GLP-1	This study
pKA486	KKA403	Surface-anchored trypsin stabilized 5×GLP-1	This study
pKA488	KKA405	Secreted wild type 5×GLP-1	This study
pKA489	KKA428	Surface-anchored wild type 5×GLP-1	This study
pKA101	KKA101	Non-expressing version of the plasmid	[24]

### Western blot

Expression of 5×GLP-1 from *Lactobacillus* was verified by Western blot. The bacterial cultures were centrifuged for 10 min at 3,000 x g when OD_600_ reached 1.0. The supernatant was filter-sterilized using a 0.2 μm filter and the pH adjusted to 7.0 with 2 M sodium hydroxide, then mixed with 2×Laemmli sample buffer (Bio-Rad) and boiled for 5 min. The pelleted cells were washed twice with PBS and resuspended in 2×Laemmli sample buffer (1/10 volume of original culture) and boiled for 10 min. The cell extract samples were centrifuged at 16,100 x g to remove cell debris and the supernatant containing soluble proteins was retained. Ten ng of purified peptide (either synthesized GLP-1 peptide or purified 5×GLP-1) was used as a positive control and 20 μl of the supernatant or cell extract were loaded onto the gel. Samples were run on a 12% SDS-polyacrylamide gel at 150 volts and the proteins were transferred onto a nitrocellulose membrane (Hybond-ECL, GE Healthcare, UK). An anti-Glucagon-like peptide-1 (Mid-molecule specific, BioPorto) mouse monoclonal antibody was used as the primary antibody at 0.5 μg/ml. Polyclonal goat anti-mouse immunoglobulin (Dako, Glostrup, Denmark) was used as secondary antibody at 1 μg/ml.

### Flow cytometry

To analyze the surface display of 5×GLP-1 from lactobacilli, 50 μl of *Lactobacillus* cultures grown in MRS to an OD_600_ of 1.0 were pelleted at 3,000 × g for 10 min and washed twice in PBS. Bacteria were incubated with anti-GLP-1 mouse monoclonal antibody (1:200) for 30 min on ice, followed by incubation with a FITC-conjugated goat anti-mouse antibody (Jackson Immunoresearch Lab., West Grove, PA, USA, 1:200) for 30 min. Antibodies were all diluted in PBS containing 1% BSA. Bacteria were washed with 1 ml PBS after each incubation. Samples were fixed in 2% phosphate buffered formaldehyde and analyzed using a FACS Calibur machine (Becton Dickinson, Franklin Lakes, NJ).

### Protein purification and *in vitro* tryptic digestion of pentameric GLP-1

Wt 5×GLP-1 and trp 5×GLP-1 positive controls were expressed in *E*. *coli* with a C-terminal His-tag and purified to approximately 85% purity (GenScript, Piscataway, NJ). The *Lactobacillus*-produced Lp wt 5×GLP-1 and Lp trp 5×GLP-1 were purified from the culture supernatant of strains (KKA405 and KKA394) with anti-E-tag monoclonal antibodies coupled to an NHS-HiTrap sepharose column (GE-healthcare) according to the manufacturer’s instructions. The eluate was concentrated to a volume of 0.5 ml using an Amicon Ultra-4 3K centrifugal filter (Millipore, Billerica, USA). The concentration of purified 5×GLP-1 was determined by the Micro BCA Protein Assay Kit (Pierce, Rockford, USA) with *E*. *coli* produced 5×GLP-1 as a standard.

Both *E*. *coli*- and *Lactobacillus*-produced 5×GLP-1 were subjected to *in vitro* trypsin digestion to mirror the *in vivo* situation. 5×GLP-1 was digested for 7 min at 37°C using a Trypsin Spin Column (TT0010, Sigma) according to the manufacturer’s instructions. Tryptic digested proteins were analyzed by silver staining before use in the *in vitro* experiments.

### Insulinotropic effect test on HIT-T15 cells

The HIT-T15 cell line (ATCC CRL-1777) was previously established from SV40 virus-transformed Syrian hamster pancreatic islet cells [30]. The cell line was a gift from Dr. Jia Yu (MMK, Karolinska Institutet). HIT-T15 cells (passages 81–82) were routinely maintained in RPMI 1640 (61870, Gibco) supplemented with 10% fetal bovine serum (FBS, 10082, Gibco) and a penicillin (100 IU/ml)/streptomycin (100 mg/ml) cocktail (Invitrogen AB, Stockholm, Sweden). The cells were cultured in T-75 culture flasks until 70% confluence, and subcultured into 6-well plates at a density of 1×10^5^ cells/well. The cells were cultured for 5 days before use in the experiment. The cells were incubated twice with 2 ml Krebs-Ringer Buffer (KRB) supplemented with 0.1% BSA at 37°C for 30 min, rinsed once with 2 ml KRB plus 0.1% BSA and incubated with 1 ml KRB buffer (either with no glucose or 5.6 mM glucose) containing digested pentameric GLP-1 purified from *E*. *coli* or lactobacilli (37°C for 60 min). One hundred nM of GLP-1-Gly8 was used as a positive control. Following incubation, 1 ml aliquots were retained from each well and centrifuged for 5 min at 1,000 × g. The cell-free supernatants (10 μl) were subjected to insulin quantification using a rat enzyme-linked immunoassay (ELISA) kit (Mercodia AB, Uppsala, Sweden).

### Animals

Male Goto-Kakizaki (GK) rats (200–250 g body weight) were used as an animal model of type 2 diabetes [31] for testing the activity of synthesized 5×GLP-1 peptide and *Lactobacillus* produced pentameric GLP-1. GK rats were bred in the department of Molecular Medicine and Surgery (Karolinska Institutet, Stockholm, Sweden). The rats were housed 3 or 4 per cage at 22°C with an alternating 12-hour light-dark cycle and were allowed free access to standard pellet diet (B&K Universal) and water. For testing the activity of *E*. *coli*-produced 5×GLP-1 peptide, rats were cannulated with a catheter inserted into the small intestine to avoid the peptide being digested before reaching the intestine (S1A Fig). The dose of intra-intestinal administration of the 5×GLP-1 peptide was determined based on preliminary experiments using the GLP-1-Gly8 (monomeric form). When GLP-1-Gly8 was given through the catheter, a reduction in blood glucose level was observed at a dose of 1 mg/kg. Considering that digestion of the 5×GLP-1 by intestinal trypsin may not be complete, 5 mg/kg body weight of 5×GLP-1 was chosen to give intra-intestinally to the rats through the catheter. For testing the *Lactobacillus* producing pentameric GLP-1, rats were gavaged with two doses of *Lactobacillus* strains (10^10^ cfu) daily. Wistar rats from a commercial breeder (Charles River) were used for islet isolation in the *in vitro* activity test of synthesized GLP-1 peptide.

### Ethics statement

All animal studies were carried out according to Karolinska Institutet guidelines of animal experiments. The animal experiment protocols were approved by the Stockholm north ethical committee (Stockholms Norra Djurförsöksetiska nämnd) (Permit Number: N 28/12). The surgery of GK rats was performed under Isoflurane anesthesia. At the end of the experiment, the rats were sedated with isoflurane before euthanasia with carbon dioxide followed by decapitation.

### Insulin secretion from rat islets

Islets from Wistar rats were used to compare the activity of GLP-1 and GLP-1-Gly8 analogue. The islets were isolated according to previously described methods [32]. The isolated islets were handpicked and maintained for 24 hours at 37°C, 5% CO_2_ in air, in RPMI 1640 culture medium with 5.5 mmol/L glucose and 10% heat-inactivated fetal bovine serum. The release of insulin was determined in static incubations. A 30-min pre-incubation in Krebs-Ringer bicarbonate buffer (KRB) solution supplemented with 2 mg/mL bovine serum albumin (fraction V, Sigma Chemical, St. Louis, Mo), 10 mmol/L 4-(2-hydroxyethyl)-1-piperazineethanesulfonic acid (HEPES), and 3.3 mmol/L glucose (37°C, pH 7.4) was performed. Batches of 3 islets were subsequently incubated for 60 min in 300 μL KRB with albumin and HEPES, as described, and in 16.7 mmol/L glucose. Insulin secretion was stimulated by co-incubation with 1, 10 and 100 nmol/L respectively of either GLP-1 or GLP-1-Gly8 and using non-stimulated islets as control. The incubations were stopped by cooling the samples on ice and insulin secretion was quantified by a radioimmunoassay [33].

### Intraperitoneal glucose tolerance test (IPGTT)

An IPGTT was used to measure the clearance of an intraperitoneally injected glucose load from the GK rat after administration of GLP-1 peptide or *Lactobacillus* expressing 5×GLP-1 [34, 35]. The IPGTT was used rather than oral glucose tolerance test (OGTT) because glucose entering in the intestine is a potent stimulator of endogenous GLP-1 secretion which could mask the effect of the exogenously administered GLP-1 peptide [36, 37].

Before the experiment, GK rats were fasted overnight, allowing access only to drinking water. A solution of glucose (2 g/kg body weight) was administered by intraperitoneal injection and blood glucose was measured at different time-points during the subsequent 3 hours. Blood was obtained by the tail-prick method and measured using a glucometer, Accu-check Aviva (Roche Diagnostic GmbH, USA).

### Oral administration of pentameric GLP-1 expressing lactobacilli to GK rats

To test the anti-diabetic effect of 5×GLP-1 expressing lactobacilli, each group (n = 6) of GK rats was given either PBS or one of the *Lactobacillus* strains KKA101, KKA394 and KKA403 by gavage twice daily (at 8:30 and 16:30) for seven days (day 0 to day 6). The lactobacilli were grown to the logarithmic phase, pelleted by centrifugation, washed twice with PBS and finally resuspended in PBS at 10^10^ cfu/ml. One ml of either *Lactobacillus* suspension or PBS was given to each rat.

Non-fasting blood glucose levels were measured by tail-prick method every morning. At the end of the experiment (the morning of day 7 after whole night fasting), rats were given the last gavage of lactobacilli and then subjected to an IPGTT with 2 g glucose/kg body weight 15 min later.

In a repeat of this experiment extended to 14 days, twenty one GK rats were randomized into 3 groups (n = 7) and given PBS, lactobacilli KKA101 and anchored 5×GLP-1 KKA403. The same protocol was followed, except that an additional serum sample was taken from the tail vein at days 0, 6 and 13. The body weight of the rats was monitored every day during the 14-day experimental period.

### Statistical analysis

Data are expressed as the mean ± SEM unless otherwise indicated. Two-way repeated measures analysis of variance (ANOVA) followed by the Student-Bonferroni multiple-range test was used to estimate the significance of differences for glycaemia between groups during the lactobacilli treatment period and IPGTT. One-way ANOVA followed by the Student-Bonferroni multiple-range test was used to estimate the significance of differences between groups for area under the curve (AUC) of blood glucose levels during IPGTT. Difference in insulinotropic effect between purified peptides from *E*. *coli* and lactobacilli was analyzed by the unpaired t-test with Welch’s correction. A value of p<0.05 was considered as statistically significant. Data were analyzed using GraphPad Prism v6.0 (GraphPad Software, San Diego California USA).

## Results and Discussion

### Synthetic monomeric GLP-1 peptides and confirmation of its bioactivity *in vitro*

**Fig 1:**
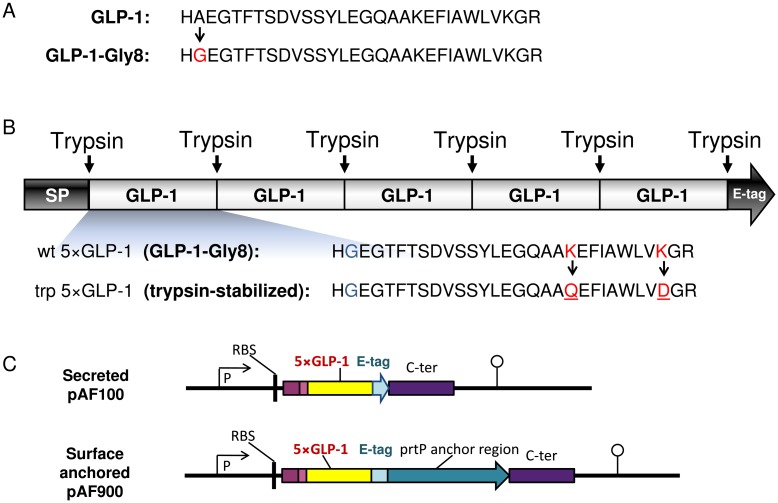
Design of GLP-1 analogues (A), pentameric GLP-1 (B) and expression cassettes for 5×GLP-1 delivery by *L*. *paracasei* (C). SP, signal peptide; RBS, ribosomal binding site; P, apf promoter; C-ter: C-terminal part of APF gene; prtP anchor translational stop codon (indicated with an arrowhead) and the transcription terminator (indicated with a lollipop).

The GLP-1 peptide and its proteolytically stabilized analogue, GLP-1-Gly8, were produced as synthetic peptides and used as controls for bioactivity of *Lactobacillus* produced GLP-1 in the subsequent experiments (Fig 1A). The insulinotropic effect of GLP-1 and GLP-1-Gly8 was assayed on pancreatic islets isolated from Wistar rats to ensure that the GLP-1-Gly8 peptide has the same bioactivity as the native molecule. Wistar rats are the progenitors of GK rats, but isolation of islets is easier than in GK rats as they have more functional islets. Both versions of the peptide stimulated insulin release in a dose-dependent way at 16.7 mmol/l glucose levels and the DPP-IV-stabilized version (GLP-1-Gly8) of the peptide had the similar capacity in stimulation of insulin release as compared to the wildtype GLP-1 peptide (S2 Table). This result confirms previous report [12] and GLP-1-Gly8 was thus selected for delivery by *Lactobacillus* in the subsequent experiments due to its longer half-life *in vivo*.

### Design of pentameric GLP-1

The N-terminal histidine of the mature peptide has previously been found to be important for the insulinotropic effect of GLP-1 [38, 39]. To be able to produce secreted GLP-1-Gly8 with an intact N-terminal by lactobacilli (starting with a histidine), 8 different signal peptides were selected for fusion to the GLP-1-Gly8 gene based on a prediction of cleavage sites by SignalP [40, 41]. However, none of them resulted in the secretion of a GLP-1-Gly8 with a correct N-terminal cleavage (S3 Table). This led us to design a pentameric GLP-1 consisting of five consecutive GLP-1-Gly8 monomers (Fig 1B). The last amino acid (glycine) of GLP-1-Gly8 was removed to create a trypsin digestion site between each monomer and in order to expose a free N-terminal histidine after digestion by the intestinal trypsin, giving a wildtype pentameric GLP-1 (wt 5×GLP-1). A trypsin-stabilized version of the pentamer (trp 5×GLP-1) was also constructed by inserting mutations at position 26 (lysine to glutamine) and 34 (lysine to aspartic acid) in the GLP-1-Gly8 monomers to stabilize it against tryptic digestion within the monomer. The pentameric GLP-1 was produced and purified from *E*. *coli* in order to obtain large amounts to be used as a positive control for testing the activity of the *Lactobacillus*-produced 5×GLP-1 in the *in vitro* and animal model.

### Construction of *Lactobacillus* expressing pentameric GLP-1

Four different expression cassettes were constructed where the wt 5×GLP-1 and the trp 5×GLP-1 were either secreted (KKA405 and KKA394) or anchored to the surface (KKA428 and KKA403) of lactobacilli (Table 1). In these cassettes, the 5×GLP-1 genes were fused to a C-terminal E-tag for easier detection and purification (Fig 1C).

**Fig 2:**
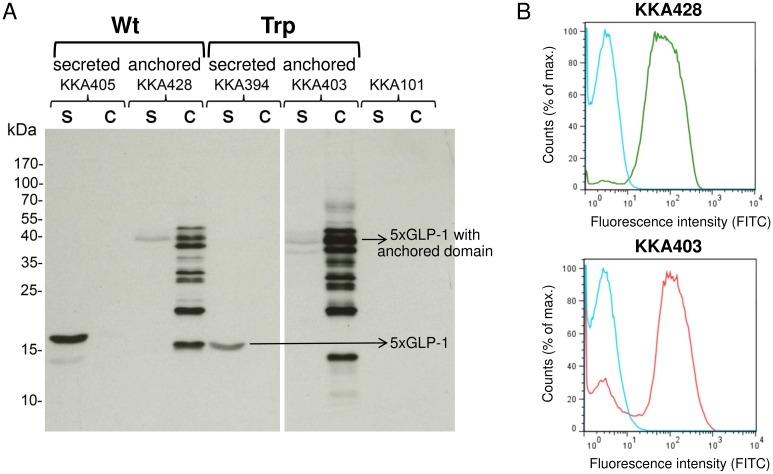
Expression and surface display of pentameric GLP-1 by transformed *L*. *paracasei* BL23. (A) Detection of 5×GLP-1 expressed by *L*. *paracasei* BL23 using Western blotting. Cell extract (c) and culture supernatant (s) of cell wall-anchored strain wt 5×GLP-1 (KKA428) and trp 5×GLP-1 (KKA403), as well as secreted strains, wt 5×GLP-1 (KKA405) and trp 5×GLP-1 (KKA394), are included. The predicted size of secreted 5×GLP-1 was 19.5 kDa (wt) and 19.4 kDa (trp) in the supernatant, whereas the anchored 5×GLP-1 fusion protein was 44.1 kDa (wt) and 44 kDa (trp) in the cell extract. (B) Surface display of anchored wt 5×GLP-1 (KKA428, green) and trp 5×GLP-1 (KKA403, red) produced by *Lactobacillus* in flow cytometry, as compared to the non-expressor strain (KKA101, blue).

Both wt 5×GLP-1 and trp 5×GLP-1 expression were successfully detected by Western blot (Fig 2A). The secreted 5×GLP-1 (19 kDa) was detected in the supernatant, whereas the 5×GLP-1 anchored fusion protein (44 kDa) was detected in the cell extract. Some 5×GLP-1 was also found in the supernatant fraction of the lactobacilli expressing the anchored construct, which is likely to be either due to saturation of anchoring sites or inefficient anchoring of 5xGLP-1. The display of anchored wt 5×GLP-1 (KKA428) and trp 5×GLP-1 (KKA403) on the surface of engineered *L*. *paracasei* BL23 cells was determined by flow cytometry (Fig 2B). Both pentameric GLP-1 proteins were displayed in equal amounts on the surface of the bacteria. The median fluorescence intensities were 29-fold increased for KKA403 and 24-fold increased for KKA428 as compared to the non-expressor *L*. *paracasei* BL23 strain (KKA101).

### *In vitro* tryptic digestion of pentameric GLP-1

**Fig 3:**
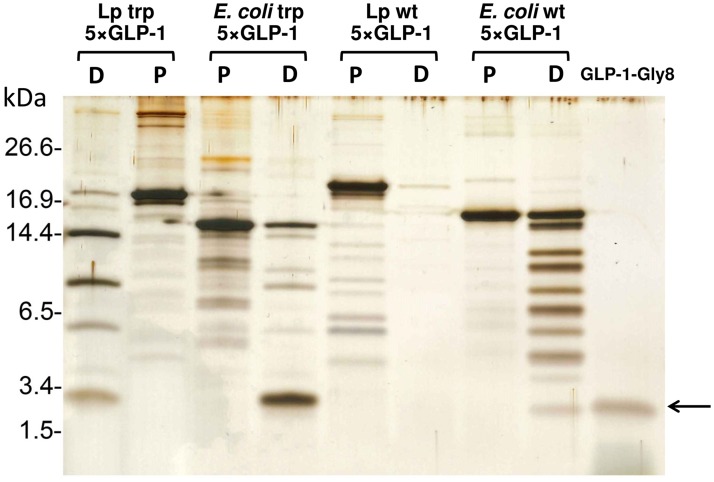
*In vitro* tryptic digestion of pentameric GLP-1 detected by Western blot. The wt 5×GLP-1 and trp 5×GLP-1 purified from *E*. *coli* and *L*. *paracasei* BL23 (KKA394 and KKA405) were digested using the trypsin spin column for 7 min. The 250 ng of purified protein (P) and 250 ng of digested protein (D) were loaded in parallel on SDS-PAGE and detected by silver staining. The arrow indicates the size of the GLP-1 monomer (3.4 kDa).

Both *E*. *coli*- and *Lactobacillus*-produced purified wt 5×GLP-1 and trp 5×GLP-1 were subjected to *in vitro* digestion in a trypsin spin column for 7 min. The digested trp 5×GLP-1 from both *E*. *coli* and *Lactobacillus* (KKA394) had a distinct banding pattern where multimeric bands containing partially digested peptides could be identified and the smallest monomeric band was the same size as the GLP-1-Gly8 control (Fig 3), indicating that the pentameric GLP-1 can be digested by trypsin into monomers and could be used for further bioactivity tests. However, the *E*. *coli* produced wt 5×GLP-1 had a different digestion pattern compared to the trp 5×GLP-1, where the wt 5×GLP-1 had a very faint band corresponding to the GLP-1 monomer and the intensity of the band did not increase when prolonging the digestion time (S2 Fig). Furthermore, the wt 5×GLP-1 from *Lactobacillus* (KKA405) disappeared completely after trypsin digestion for 7 min and there was no band corresponding to the GLP-1 monomer (Fig 3). This is probably due to internal trypsin-sensitive sites of the wt 5×GLP-1 being cut and the oligopeptides generated were not detectable.

### Insulinotropic effect of pentameric GLP-1 on HIT-T15 cells

**Fig 4:**
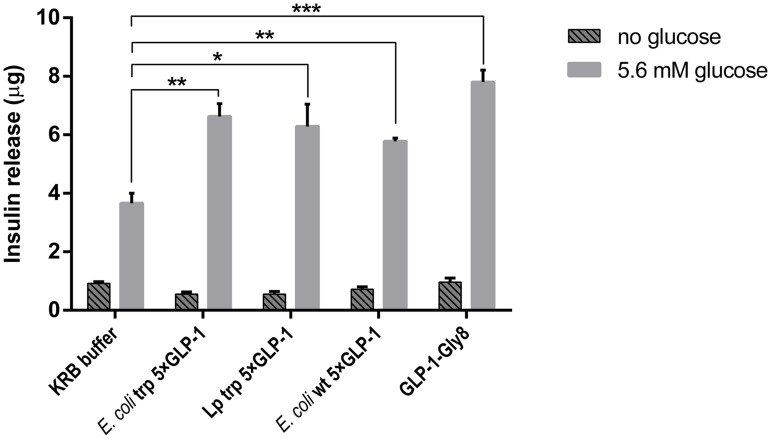
Insulin release by HIT-T15 cells in the presence of digested pentameric GLP-1. HIT-T15 cells were incubated with 1 ml Krebs-Ringer Buffer (KRB) supplemented either without glucose or with 5.6 mM glucose, containing digested 5×GLP-1 (100 nm before digestion) purified from *E*. *coli* or *L*. *paracasei* BL23 (37°C for 1 h). KRB buffer alone was used as a baseline control. 100 nm GLP-1-Gly8 was used as a positive control. The insulin level is presented as mean ± SD (n = 3). ***p<0.001, **p<0.01, *p<0.05, unpaired *t*-test with Welch’s correction.

The hamster β-cell line HIT-T15 is one of the most extensively studied beta-cell-like *in vitro* models and exhibit glucose-stimulated insulin secretion [30]. HIT-T15 cells were used to test the insulinotropic effect of *Lactobacillus* (KKA394) secreted trp 5×GLP-1 (after trypsin digestion). The *E*. *coli* produced wt 5×GLP-1 and trp 5×GLP-1 (after trypsin digestion) were also tested for comparison. As shown in Fig 4, there was no difference in insulin secretion between the control and digested *E*. *coli* or *Lactobacillus* trp 5×GLP-1 (100 nM) without glucose supplementation. The presence of 5.6 mM glucose significantly increased the insulinotropic effect of GLP-1 (p<0.01 for Lp trp 5×GLP-1, unpaired t-test with Welch’s correction). The insulin secretion caused by the two types of *E*. *coli* produced 5×GLP-1 and the *Lactobacillus* produced trp 5×GLP-1 (after trypsin digestion) was significantly higher than the KRB buffer control, almost equivalent to the synthetic GLP-1-Gly8 positive control (Fig 4). *Lactobacillus*-produced wt 5×GLP-1 was not tested since no monomeric GLP-1 could be detected after trypsin digestion (Fig 3). Therefore, we did not include *Lactobacillus* produced wt 5×GLP-1 in subsequent experiments.

### Activity of synthetic GLP-1-Gly8 peptide *in vivo* by IPGTT

GK rat is a substrain of Wistar rat that spontaneously develops adult onset type 2 diabetes early in life. It is considered to be one of the best characterized non-obese animal models of type 2 diabetes [42] since they exhibit similar metabolic, hormonal, and vascular changes as observed in the human disease [31].

**Fig 5:**
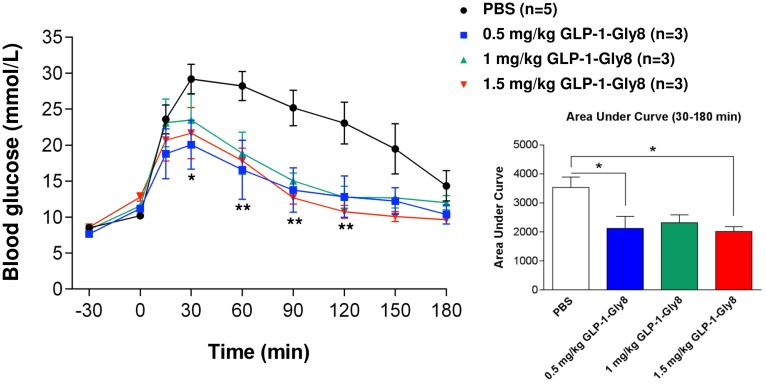
Bioactivity of GLP-1-Gly8 injected subcutaneously using IPGTT. Blood glucose levels in the intraperitoneal glucose tolerance test (IPGTT) in GK rats. GLP-1-Gly8 was given subcutaneously at 0.5 mg/kg (blue), 1 mg/kg (green) or 1.5 mg/kg (red) body weight 30 min prior to the glucose challenge test (Glucose: 2 g/kg of body weight). The area under the curve (AUC) for glucose levels corresponding to 30–180 min is shown. Data are presented as the mean ± SEM (PBS group n = 5, others n = 3). **p<0.01, *p<0.05 versus PBS.

The bioactivity of the monomeric synthetic GLP-1-Gly8 peptide was verified in GK rats using IPGTT. GLP-1-Gly8 was given subcutaneously at a dose of 0.5–1.5 mg/kg body weight 30 min prior to the glucose tolerance test. As shown in Fig 5, the blood glucose levels at 30, 60, 90 and 120 min were significantly decreased compared to the PBS control when 0.5 mg/kg body weight of GLP-1-gly8 was given. Higher doses of GLP-1-Gly8 peptide also decrease the blood glucose levels significantly (from 60 to 120 min and from 60 to 180 min for 1 mg/kg and 1.5 mg/kg GLP-1-Gly8 doses, respectively) (F (3, 10) = 3.776, p = 0.0478, two-way repeated measures ANOVA followed by Bonferroni’s multiple comparison test). A significant reduction of the area under blood glucose curve was also observed between 30 and 180 min (F (3, 10) = 4.906, p = 0.0239, one-way ANOVA followed by Bonferroni’s multiple comparison test), suggesting that GLP-1-Gly8 could be used instead of native GLP-1 as a glucose-dependent insulinotropic agent in the GK rat model (Fig 5).

### Activity of *E*. *coli* produced pentameric GLP-1 *in vivo* by IPGTT

To verify the bioactivity of pentameric GLP-1, GK rats were initially fed with *E*. *coli*-produced pentameric GLP-1 through a catheter inserted into the small intestine, and the glucose level was monitored for 3 hours after an intraperitoneal injection of glucose. The pentameric GLP-1 is expected to be digested by the intestinal trypsin into five active monomers of GLP-1 when it is released into the intestine. The purpose of using an intestinal catheter was to avoid the peptide being digested by other proteases before reaching the intestine.

**Fig 6:**
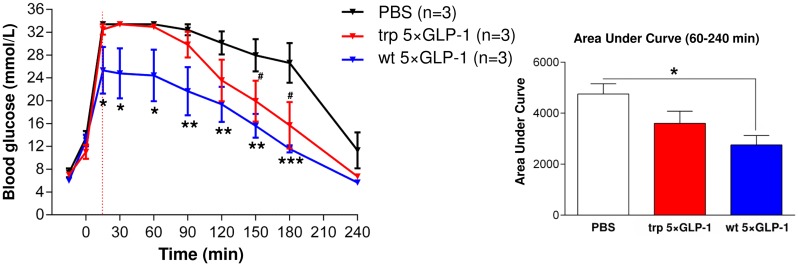
Bioactivity of pentameric GLP-1 using IPGTT. Blood glucose levels in the intraperitoneal glucose tolerance test (IPGTT) in GK rats. *E*. *coli*-produced 5×GLP-1 was administrated intra-intestinally at 5 mg/kg body weight to GK rats 15 min prior to the glucose challenge (glucose: 3 g/kg of body weight). The AUC for the period corresponding to 60–240 min is shown. Data are presented as the mean ± SEM (n = 3). ***p<0.001, **p<0.01, *p<0.05 versus PBS; ^#^p<0.05 versus PBS.

The blood glucose level of rats receiving wt 5×GLP-1 at 5 mg/kg body weight was significantly lower from 15 to 180 min as compared to rats receiving PBS (F (2, 6) = 5.570, p = 0.0429, two-way repeated measures ANOVA followed by Bonferroni’s multiple comparison test). The area under the glucose curve was also significantly reduced from 60 to 240 min after intraperitoneal injection of glucose (3 g/kg of bodyweight) for the rats receiving wt 5×GLP-1 as compared to rats receiving PBS only (F (2, 6) = 5.821, p = 0.0393, one-way ANOVA followed by Bonferroni’s multiple comparison test) (Fig 6). The blood glucose level of rats receiving trp 5×GLP-1 was also significantly lower at 150 and 180 min (P<0.05) with a decreased area under the glucose curve, although not to a statistically significant degree (P>0.05), as compared to that measured in response to the PBS control (Fig 6). The flat blood glucose stimulation curve (no stimulation peak for PBS and trp 5×GLP-1 treatments from 15 to 60 min) is due to the upper limit of the glucometer used. The experiment with wt 5×GLP-1 was repeated with more animals (n = 7) where glucose was given at a lower dose (2 g/kg of bodyweight) to the rats (S1B Fig). A similar trend in lowering the blood glucose level was observed, but not to a statistically significant degree as compared to the PBS control (a significant reduction in area under curve between 30–90 min, p = 0.0456; but no significant difference in area under curve between 30–240 min, p>0.05, unpaired t-test with Welch’s correction).

### *In vivo* delivery of pentameric GLP-1 by *Lactobacillus* to diabetic rats

The antidiabetic effect of pentameric GLP-1 was investigated in GK rats after oral administration of *Lactobacillus* expressing trp 5×GLP-1 or a negative control consisting of *Lactobacillus* containing an empty expression plasmid (KKA101).

**Fig 7:**
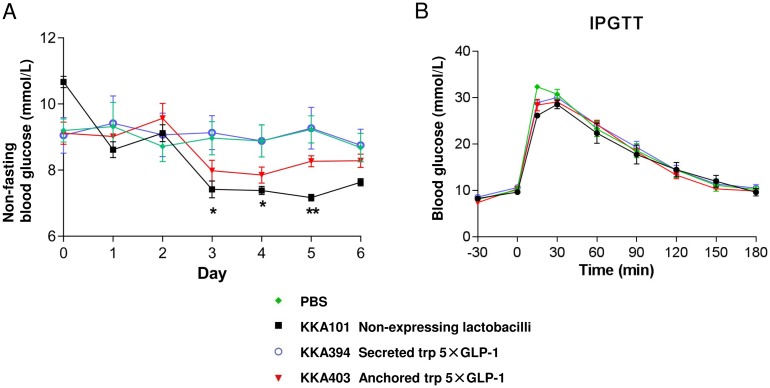
Feeding GK rats with pentameric GLP-1 expressing *Lactobacillus*. (A) Daily non-fasting blood glucose level during the 7-day *Lactobacillus* feeding experiment. The rats received 10^10^ cfu of different *Lactobacillus* strains (KKA101, KKA394 and KKA403) or PBS twice daily by gavage until the end of the study. (B) Blood glucose level during an IPGTT after 7 days feeding experiment. The rats were given the last gavage of lactobacilli followed by an IPGTT with 2 g/kg body weight of glucose. The blood glucose level is presented as mean ± SEM (n = 6). **p<0.01, *p<0.05 versus PBS.

During the 7-day feeding experiment, non-fasting blood glucose levels were measured every morning and no significant difference was observed among groups during the treatment except that the rats receiving KKA101 have a significant decrease in blood glucose during day 3 to 5 as compared to the PBS control (F (3, 20) = 1.233, p = 0.3237, two-way repeated measures ANOVA followed by Bonferroni’s multiple comparison test) (Fig 7A). *Lactobacillus* expressing surface anchored trp 5×GLP-1 (KKA403) showed a similar trend to lower the blood glucose level, but not to a statistically significant degree (p>0.05). The trp 5×GLP-1 secreting *Lactobacillus* (KKA394) generated the same blood glucose level compared to the PBS negative control. Rats were subjected to IPGTT at the end of the experiment and no significant difference was observed between groups receiving different lactobacilli or PBS (area under the curve, F (3, 20) = 0.1137, p = 0.9511, one-way ANOVA followed by Bonferroni’s multiple comparison test) (Fig 7B and S3A Fig). This suggests that short term oral feeding of 5×GLP-1 expressing lactobacilli does not have an immediate effect in reducing hyperglycemia in GK rats.

**Fig 8:**
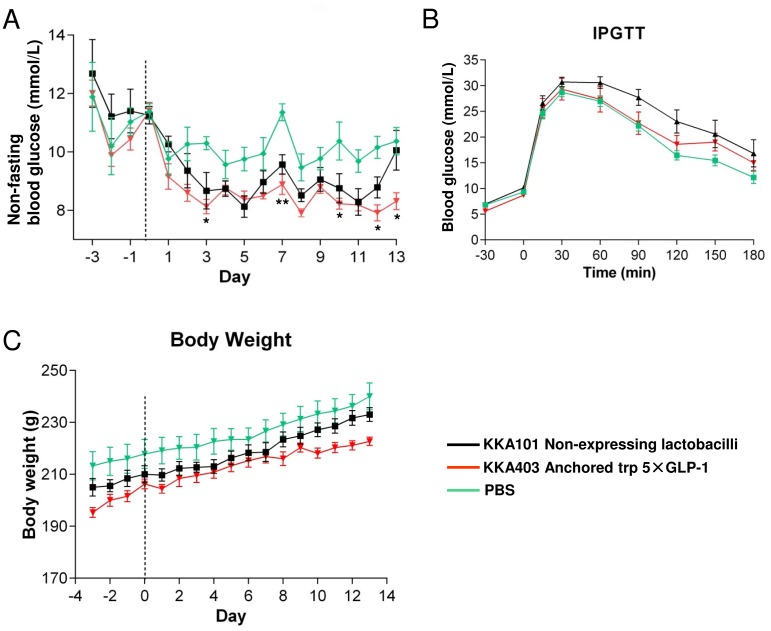
Effect of feeding GK rats with pentameric GLP-1 expressing *Lactobacillus* on blood glucose level, IPGTT and body weight. (A) Daily non-fasting blood glucose level during the 14-day *Lactobacillus* feeding experiment. (B) Blood glucose level during an IPGTT after 14 days feeding experiment. Glucose: 2 g/kg of body weight. (C) Body weight was monitored during the 14-day study. Data are presented as mean ± SEM (n = 7). **p<0.01, *p<0.05 between KKA403 and PBS.

The treatment with *Lactobacillus* expressing secreted trp 5×GLP-1 depends on the secretion of the peptide *in situ* in the intestinal tract. On the other hand, the *Lactobacillus* expressing anchored trp 5×GLP-1 also acts like beads already loaded with GLP-1 peptides that can be immediately proteolytically released upon contact with trypsin in the intestine. Thus, *in situ* secretion depends on the growth of the lactobacilli in the intestine and might lead to suboptimal production rates and a decreased effect compared to surface anchored expression of GLP-1 when growth conditions are limiting the secretion of GLP-1. A 14-day animal experiment was therefore performed to investigate if the trend in glucose level reduction observed with KKA101 and KKA403 could be improved by extending the feeding duration to two weeks (Fig 8). Non-fasting blood glucose levels of the rats receiving KKA101 (non-expressor) and KKA403 (anchored trp 5×GLP-1) treatment were decreased as compared to the PBS control from day 3 until the end of the experiment, although the difference was only statistically significant between KKA403 and PBS (at day 3, 7, 10, 12 and 13). However, there was no significant difference between KKA403 and non-expressor strain KKA101 (F (2, 18) = 6.544, p = 0.0073, two-way repeated measures ANOVA followed by Bonferroni’s multiple comparison test) (Fig 8A).

GLP-1 was previously shown to reduce basal food intake and body weight, however the body weight gain (F (2, 18) = 0.0926, p = 0.9118, one-way ANOVA followed by Bonferroni’s multiple comparison test) of the GK rats monitored every day during the 14-day experimental period did not differ significantly between the groups (Fig 8C). IPGTT was also performed at the last day of the experiment and, similar to the 7-day animal experiment, there was no significant difference between lactobacilli and PBS groups (area under the glucose curve, F (2, 18) = 2.337, p = 0.1252, one-way ANOVA followed by Bonferroni’s multiple comparison test) (Fig 8B and S3B Fig).

The following points should be considered when interpreting the results of the rat feeding experiment: First and foremost, the limited amount of GLP-1 delivered to the intestine might be the main reason that no significant difference was observed between KKA403 and the non-expressor control lactobacilli. If monomeric GLP-1-anchored lactobacilli were designed from the beginning, the theoretical maximum number of molecules expected to be anchored on the bacterial surface is 6 x 10^3^ [27]. 10^10^ cfu bacteria were administered per rat, yielding a total of 0.3 μg GLP-1 per rat. This is significantly lower than the amount of purified *E*. *coli* 5×GLP-1 (around 1 mg/rat) that was given through the intestinal catheter (although not a titrated minimal dose). Therefore, we have designed pentameric GLP-1 that could, in theory, release 5 monomers of GLP-1 after intestinal trypsin digestion to increase the amount of GLP-1 that each *Lactobacillus* could deliver. We also expect a higher local concentration of 5×GLP-1 in the intestine when delivered by *Lactobacillus*, since lactobacilli delivery gives a constitutive expression of peptide. Even so, improving the expression level of our *Lactobacillus* delivery system is still required to see an effect.

Secondly, it should be also noted that not all lactobacilli will retain their ability to express 5×GLP-1 when colonizing in the intestine. The plasmid encoding 5×GLP-1, which is maintained by antibiotic presence in the culture medium, might be lost due to lack of selective pressure in the gastro-intestinal (GI) tract. To evaluate the rate of plasmid loss during transit through the GI tract of GK rat, we generated a spontaneously rifampicin-resistant strain carrying the GLP-1-expressing plasmid. The plasmid loss rate was approximately 40% for the anchored trp 5×GLP-1 strains (carrying pKA486) and 65% for the secreted 5×GLP-1 (carrying pKA480) strains (unpublished data). This is acceptable for our feeding experiment since lactobacilli were given twice per day at large doses. However, further experiments should be performed to improve the 5×GLP-1 delivery by integrating the genes into the *Lactobacillus* chromosome and thereby stabilizing its expression [27].

During the preparation of our manuscript, Agarwal *et al*. described oral delivery of GLP-1 by *Lactococcus lactis* [43] and showed a decrease in blood glucose levels of 10–20% at 5–8 h following administration of *L*. *lactis* secreting GLP-1 in Zucker diabetic fatty (ZDF) rats, which returned to baseline after 11 h. However, additional N-terminal amino acids were introduced in the GLP-1 peptide due to the cloning strategy and the long term effect of *Lactococcus* feeding on glucose level was not measured. *Lactococcus* has previously been shown to be one of the most suitable lactic acid bacteria (LAB) strains for expression of foreign proteins [44, 45]. However, *Lactococcus* does not colonize the intestinal tract and its effect on the intestinal microbial balance is unknown. In contrast, lactobacilli colonize the gut to a greater extent and have been proven to have an anti-diabetic potential in rodents, as demonstrated by many groups [46–48]. A recent paper also reported that *L*. *reuteri* improves incretin and insulin secretion in glucose-tolerant humans [49]. According to Panwar et al., different *Lactobacillus* strains including *L*. *rhamnosus* GG, *L*. *casei*, *L*. *acidophilus*, *L*. *plantarum* and *L*. *reuteri* have all been shown to either lower plasma glucose levels or to improve insulin resistance in diabetic mice or rat models [50]. Our one week feeding experiment also showed that non-expressor *L*. *paracasei* could significantly lower the blood glucose level during the feeding period, which indicates that lactobacilli themselves could be considered as a possible therapeutic candidate for type 2 diabetes.

As was evident in the *in vitro* insulinotropic assay, trp 5×GLP-1 (after trypsin digestion) purified from the supernatant of trp 5×GLP-1-secreting lactobacilli was effective in stimulation of insulin secretion from HIT-T15 cells. Nevertheless, only the trp 5×GLP-1-anchored lactobacilli and non-expressor lactobacilli demonstrated a trend towards reduction of blood glucose levels in the lactobacilli feeding experiment. This is likely due to an undefined mechanism whereby surface molecules involved in the anti-diabetic effect of lactobacilli had been hindered in the secreting-strain, but not in the surface anchored-strain. This interesting observation might lead to future studies investigating the anti-diabetic effect of *Lactobacillus* strains.

## Conclusion

We have previously expressed antibody fragments against selected pathogens in lactobacilli for prevention of infectious diseases [27, 28]. In the present study, we have expanded our *Lactobacillus* delivery system for therapeutic peptide (GLP-1) delivery. We could show that a pentameric GLP-1 (protease cleavable oligomers) can be expressed in both the secreted form and anchored to the cell wall by lactobacilli, and retains its *in vitro* bioactivity following digestion by intestinal trypsin. However, in order to be able to induce a significant insulinotropic effect in diabetic animal models, further work needs to be carried out to increase the expression level of GLP-1 by lactobacilli.

## Supporting Information

S1 Fig *In vivo* bioactivity test of pentameric GLP-1 administered through an intestinal catheter. (A) The small intestine of GK rats was cannulated with a catheter to facilitate administration of the 5×GLP-1 peptide intra-intestinally. (B) Wt 5×GLP-1 was administered intra-intestinally at 5 mg/kg body weight to GK rats 30 min prior to the glucose challenge (glucose: 2 g/kg of body weight). The area under the curve (AUC) for glucose levels for the period corresponding to 30 to 240 min is shown. Data are presented as the mean ± SEM (PBS group, n = 4; 5×GLP-1 group, n = 7). *p<0.05 versus PBS, unpaired t-test with Welch’s correction.
(TIF)

S2 Fig *In vitro* tryptic digestion of wt 5×GLP-1 by trypsin spin column. The wt 5×GLP-1 purified from *E*. *coli* was digested using a trypsin spin column for 1, 2, 4 or 6 hours. The purified protein (2.5 μg) and digested protein (2.5 μg) were loaded in parallel on a SDS-PAGE gel and detected by Coomassie stain. The arrow indicates the size of GLP-1 monomer (3.4kDa).
(TIF)

S3 Fig Area under curve during IPGTT at the end of *in vivo* lactobacilli feeding experiment. (A) The area under the curve (AUC) for glucose levels for the period corresponding to 30–180 min after the 7-day feeding experiment (n = 6). (B) The AUC for glucose levels for the period corresponding to 30–180 min after the 14-day feeding experiment (n = 7). Data are presented as the mean ± SEM.
(TIF)

S1 Table Synthetic genes used for plasmid construction. (DOCX)

S2 Table Effects of GLP-1 peptides on insulin release from isolated rat pancreatic islets. (DOCX)

S3 Table Signal peptides tested for secretion of GLP-1 peptide with right N-terminal cleavage. (DOCX)

## References

[1] Kumar V, Abbas AK, Fausto N, Robbins SL, Cotran RS. Robbins and Cotran pathologic basis of disease. Philadelphia: Elsevier Saunders; 2005.

[2] Nauck MA. Is glucagon-like peptide 1 an incretin hormone? Diabetologia. 1999;42(3):373–9. 10.1007/s001250051165 . 10096792

[3] Shirazi R, Palsdottir V, Collander J, Anesten F, Vogel H, Langlet F, et al Glucagon-like peptide 1 receptor induced suppression of food intake, and body weight is mediated by central IL-1 and IL-6. Proceedings of the National Academy of Sciences of the United States of America. 2013;110(40):16199–204. 10.1073/pnas.1306799110 24048027 PMC3791711

[4] Holst JJ. The physiology of glucagon-like peptide 1. Physiological reviews. 2007;87(4):1409–39. Epub 2007/10/12. 10.1152/physrev.00034.2006 . 17928588

[5] Arulmozhi DK, Portha B. GLP-1 based therapy for type 2 diabetes. European journal of pharmaceutical sciences: official journal of the European Federation for Pharmaceutical Sciences. 2006;28(1–2):96–108. 10.1016/j.ejps.2006.01.003 . 16488579

[6] Levy JC. Therapeutic intervention in the GLP-1 pathway in Type 2 diabetes. Diabetic Medicine. 2006;23:14–9. 10.1111/j.1464-5491.2006.01833e.x 16483260

[7] Chia CW, Egan JM. Incretin-based therapies in type 2 diabetes mellitus. The Journal of clinical endocrinology and metabolism. 2008;93(10):3703–16. 10.1210/jc.2007-2109 18628530 PMC2579648

[8] Fehmann HC, Göke B. The Insulinotropic Gut Hormone Glucagon-like Peptide-1: Karger; 1997.

[9] Hui H, Farilla L, Merkel P, Perfetti R. The short half-life of glucagon-like peptide-1 in plasma does not reflect its long-lasting beneficial effects. European journal of endocrinology / European Federation of Endocrine Societies. 2002;146(6):863–9. . 12039708 10.1530/eje.0.1460863

[10] Bond A. Exenatide (Byetta) as a novel treatment option for type 2 diabetes mellitus. Proceedings. 2006;19(3):281–4. 17252050 10.1080/08998280.2006.11928181 PMC1484540

[11] Garber AJ. Long-acting glucagon-like peptide 1 receptor agonists: a review of their efficacy and tolerability. Diabetes care. 2011;34 Suppl 2:S279–84. 10.2337/dc11-s231 21525469 PMC3632193

[12] Burcelin R, Dolci W, Thorens B. Long-lasting antidiabetic effect of a dipeptidyl peptidase IV-resistant analog of glucagon-like peptide-1. Metabolism: clinical and experimental. 1999;48(2):252–8. Epub 1999/02/19. . 10024091 10.1016/s0026-0495(99)90043-4

[13] Steinert RE, Poller B, Castelli MC, Friedman K, Huber AR, Drewe J, et al Orally Administered Glucagon-Like Peptide-1 Affects Glucose Homeostasis Following an Oral Glucose Tolerance Test in Healthy Male Subjects. Clinical Pharmacology & Therapeutics. 2009;86(6):644–50. 10.1038/clpt.2009.159 19727071

[14] Araujo F, Fonte P, Santos HA, Sarmento B. Oral delivery of glucagon-like peptide-1 and analogs: alternatives for diabetes control? Journal of diabetes science and technology. 2012;6(6):1486–97. Epub 2013/01/09. 23294796 10.1177/193229681200600630 PMC3570891

[15] Youn YS, Chae SY, Lee S, Kwon MJ, Shin HJ, Lee KC. Improved peroral delivery of glucagon-like peptide-1 by site-specific biotin modification: design, preparation, and biological evaluation. European journal of pharmaceutics and biopharmaceutics: official journal of Arbeitsgemeinschaft fur Pharmazeutische Verfahrenstechnik eV. 2008;68(3):667–75. 10.1016/j.ejpb.2007.07.009 . 17904340

[16] Chae SY, Jin CH, Shin HJ, Youn YS, Lee S, Lee KC. Preparation, characterization, and application of biotinylated and biotin-PEGylated glucagon-like peptide-1 analogues for enhanced oral delivery. Bioconjugate chemistry. 2008;19(1):334–41. 10.1021/bc700292v . 18078308

[17] Joseph JW, Kalitsky J, St-Pierre S, Brubaker PL. Oral delivery of glucagon-like peptide-1 in a modified polymer preparation normalizes basal glycaemia in diabetic db/db mice. Diabetologia. 2000;43(10):1319–28. 10.1007/s001250051529 . 11079752

[18] Nguyen HN, Wey SP, Juang JH, Sonaje K, Ho YC, Chuang EY, et al The glucose-lowering potential of exendin-4 orally delivered via a pH-sensitive nanoparticle vehicle and effects on subsequent insulin secretion in vivo. Biomaterials. 2011;32(10):2673–82. 10.1016/j.biomaterials.2010.12.044 . 21256586

[19] Wells JM, Mercenier A. Mucosal delivery of therapeutic and prophylactic molecules using lactic acid bacteria. Nature reviews Microbiology. 2008;6(5):349–62. 10.1038/nrmicro1840 . 18345021 PMC7096801

[20] Tabuchi M, Ozaki M, Tamura A, Yamada N, Ishida T, Hosoda M, et al Antidiabetic effect of Lactobacillus GG in streptozotocin-induced diabetic rats. Bioscience, biotechnology, and biochemistry. 2003;67(6):1421–4. 10.1271/bbb.67.1421 . 12843677

[21] Yun SI, Park HO, Kang JH. Effect of Lactobacillus gasseri BNR17 on blood glucose levels and body weight in a mouse model of type 2 diabetes. Journal of applied microbiology. 2009;107(5):1681–6. 10.1111/j.1365-2672.2009.04350.x . 19457033

[22] Duan FF, Liu JH, March JC. Engineered commensal bacteria reprogram intestinal cells into glucose-responsive insulin-secreting cells for the treatment of diabetes. Diabetes. 2015;64(5):1794–803. Epub 2015/01/30. 10.2337/db14-0635 25626737 PMC4407861

[23] Gunaydin G, Alvarez B, Lin Y, Hammarstrom L, Marcotte H. Co-expression of anti-rotavirus proteins (llama VHH antibody fragments) in Lactobacillus: development and functionality of vectors containing two expression cassettes in tandem. PloS one. 2014;9(4):e96409 10.1371/journal.pone.0096409 24781086 PMC4004553

[24] Andersen KK, Marcotte H, Alvarez B, Boyaka PN, Hammarstrom L. In situ gastrointestinal protection against anthrax edema toxin by single-chain antibody fragment producing lactobacilli. BMC Biotechnol. 2011;11:126 Epub 2011/12/22. 10.1186/1472-6750-11-126 22185669 PMC3295704

[25] Maze A, Boel G, Zuniga M, Bourand A, Loux V, Yebra MJ, et al Complete genome sequence of the probiotic Lactobacillus casei strain BL23. Journal of bacteriology. 2010;192(10):2647–8. 10.1128/JB.00076-10 20348264 PMC2863562

[26] Acedo-Felix E, Perez-Martinez G. Significant differences between Lactobacillus casei subsp. casei ATCC 393T and a commonly used plasmid-cured derivative revealed by a polyphasic study. International journal of systematic and evolutionary microbiology. 2003;53(Pt 1):67–75. Epub 2003/03/27. 10.1099/ijs.0.02325-0 . 12656154

[27] Martin MC, Pant N, Ladero V, Gunaydin G, Andersen KK, Alvarez B, et al Integrative expression system for delivery of antibody fragments by lactobacilli. Applied and environmental microbiology. 2011;77(6):2174–9. 10.1128/AEM.02690-10 21257814 PMC3067310

[28] Kruger C, Hu Y, Pan Q, Marcotte H, Hultberg A, Delwar D, et al In situ delivery of passive immunity by lactobacilli producing single-chain antibodies. Nature biotechnology. 2002;20(7):702–6. 10.1038/nbt0702-702 . 12089555

[29] Marcotte H, Koll-Klais P, Hultberg A, Zhao Y, Gmur R, Mandar R, et al Expression of single-chain antibody against RgpA protease of Porphyromonas gingivalis in Lactobacillus. Journal of applied microbiology. 2006;100(2):256–63. 10.1111/j.1365-2672.2005.02786.x . 16430501

[30] Santerre RF, Cook RA, Crisel RM, Sharp JD, Schmidt RJ, Williams DC, et al Insulin synthesis in a clonal cell line of simian virus 40-transformed hamster pancreatic beta cells. Proceedings of the National Academy of Sciences of the United States of America. 1981;78(7):4339–43. 6270673 10.1073/pnas.78.7.4339 PMC319785

[31] Ostenson CG, Efendic S. Islet gene expression and function in type 2 diabetes; studies in the Goto-Kakizaki rat and humans. Diabetes, obesity & metabolism. 2007;9 Suppl 2:180–6. 10.1111/j.1463-1326.2007.00787.x . 17919192

[32] Ostenson CG, Grill V. Differences in long-term effects of L-glutamine and D-glucose on insulin release from rat pancreatic islets. Molecular and cellular endocrinology. 1986;45(2–3):215–21. . 3086158 10.1016/0303-7207(86)90150-4

[33] Herbert V, Lau KS, Gottlieb CW, Bleicher SJ. Coated charcoal immunoassay of insulin. The Journal of clinical endocrinology and metabolism. 1965;25(10):1375–84. 10.1210/jcem-25-10-1375 . 5320561

[34] Yassin K, Huyen VT, Hoa KN, Ostenson CG. Herbal extract of gynostemma pentaphyllum decreases hepatic glucose output in type 2 diabetic goto-kakizaki rats. International journal of biomedical science: IJBS. 2011;7(2):131–6. Epub 2011/06/01. 23675229 PMC3614830

[35] Muller C, Yassin K, Li LS, Palmblad M, Efendic S, Berggren PO, et al ARA290 improves insulin release and glucose tolerance in type 2 diabetic GK rats. Molecular medicine (Cambridge, Mass). 2015 Epub 2016/01/07. 10.2119/molmed.2015.00267 26736179 PMC4818260

[36] Chaikomin R, Doran S, Jones KL, Feinle-Bisset C, O'Donovan D, Rayner CK, et al Initially more rapid small intestinal glucose delivery increases plasma insulin, GIP, and GLP-1 but does not improve overall glycemia in healthy subjects. American journal of physiology Endocrinology and metabolism. 2005;289(3):E504–7. Epub 2005/05/12. 10.1152/ajpendo.00099.2005 . 15886226

[37] Kuo P, Chaikomin R, Pilichiewicz A, O'Donovan D, Wishart JM, Meyer JH, et al Transient, early release of glucagon-like peptide-1 during low rates of intraduodenal glucose delivery. Regulatory peptides. 2008;146(1–3):1–3. Epub 2007/10/30. 10.1016/j.regpep.2007.09.032 . 17964673

[38] Suzuki S, Kawai K, Ohashi S, Mukai H, Yamashita K. Comparison of the effects of various C-terminal and N-terminal fragment peptides of glucagon-like peptide-1 on insulin and glucagon release from the isolated perfused rat pancreas. Endocrinology. 1989;125(6):3109–14. Epub 1989/12/01. 10.1210/endo-125-6-3109 . 2684616

[39] Donnelly D. The structure and function of the glucagon-like peptide-1 receptor and its ligands. British journal of pharmacology. 2012;166(1):27–41. 10.1111/j.1476-5381.2011.01687.x 21950636 PMC3415635

[40] Petersen TN, Brunak S, von Heijne G, Nielsen H. SignalP 4.0: discriminating signal peptides from transmembrane regions. Nature methods. 2011;8(10):785–6. 10.1038/nmeth.1701 . 21959131

[41] Mathiesen G, Sveen A, Brurberg MB, Fredriksen L, Axelsson L, Eijsink VG. Genome-wide analysis of signal peptide functionality in Lactobacillus plantarum WCFS1. BMC genomics. 2009;10:425 10.1186/1471-2164-10-425 19744343 PMC2748100

[42] Akash MS, Rehman K, Chen S. Goto-Kakizaki rats: its suitability as non-obese diabetic animal model for spontaneous type 2 diabetes mellitus. Current diabetes reviews. 2013;9(5):387–96. . 23855509 10.2174/15733998113099990069

[43] Agarwal P, Khatri P, Billack B, Low WK, Shao J. Oral delivery of glucagon like peptide-1 by a recombinant Lactococcus lactis. Pharmaceutical research. 2014;31(12):3404–14. 10.1007/s11095-014-1430-3 . 24928365

[44] Steidler L, Neirynck S, Huyghebaert N, Snoeck V, Vermeire A, Goddeeris B, et al Biological containment of genetically modified Lactococcus lactis for intestinal delivery of human interleukin 10. Nature biotechnology. 2003;21(7):785–9. 10.1038/nbt840 . 12808464

[45] Steidler L, Hans W, Schotte L, Neirynck S, Obermeier F, Falk W, et al Treatment of murine colitis by Lactococcus lactis secreting interleukin-10. Science. 2000;289(5483):1352–5. . 10958782 10.1126/science.289.5483.1352

[46] Naito E, Yoshida Y, Makino K, Kounoshi Y, Kunihiro S, Takahashi R, et al Beneficial effect of oral administration of Lactobacillus casei strain Shirota on insulin resistance in diet-induced obesity mice. Journal of applied microbiology. 2011;110(3):650–7. 10.1111/j.1365-2672.2010.04922.x . 21281408

[47] Hsieh FC, Lee CL, Chai CY, Chen WT, Lu YC, Wu CS. Oral administration of Lactobacillus reuteri GMNL-263 improves insulin resistance and ameliorates hepatic steatosis in high fructose-fed rats. Nutrition & metabolism. 2013;10(1):35 10.1186/1743-7075-10-35 23590862 PMC3637306

[48] Lin CH, Lin CC, Shibu MA, Liu CS, Kuo CH, Tsai FJ, et al Oral Lactobacillus reuteri GMN-32 treatment reduces blood glucose concentrations and promotes cardiac function in rats with streptozotocin-induced diabetes mellitus. The British journal of nutrition. 2014;111(4):598–605. 10.1017/S0007114513002791 . 24001238

[49] Simon MC, Strassburger K, Nowotny B, Kolb H, Nowotny P, Burkart V, et al Intake of Lactobacillus reuteri Improves Incretin and Insulin Secretion in Glucose-Tolerant Humans: A Proof of Concept. Diabetes care. 2015;38(10):1827–34. Epub 2015/06/19. 10.2337/dc14-2690 . 26084343

[50] Panwar H, Rashmi HM, Batish VK, Grover S. Probiotics as potential biotherapeutics in the management of type 2 diabetes—prospects and perspectives. Diabetes/metabolism research and reviews. 2013;29(2):103–12. Epub 2012/12/12. 10.1002/dmrr.2376 . 23225499

