# Supplementary figures and images for: Oral Delivery of Pentameric Glucagon-Like Peptide-1 by Recombinant Lactobacillus in Diabetic Rats
Source: PLoS One. 2016 Sep 9;11(9):e0162733. doi: 10.1371/journal.pone.0162733 (PMC5017604; doi:10.1371/journal.pone.0162733)

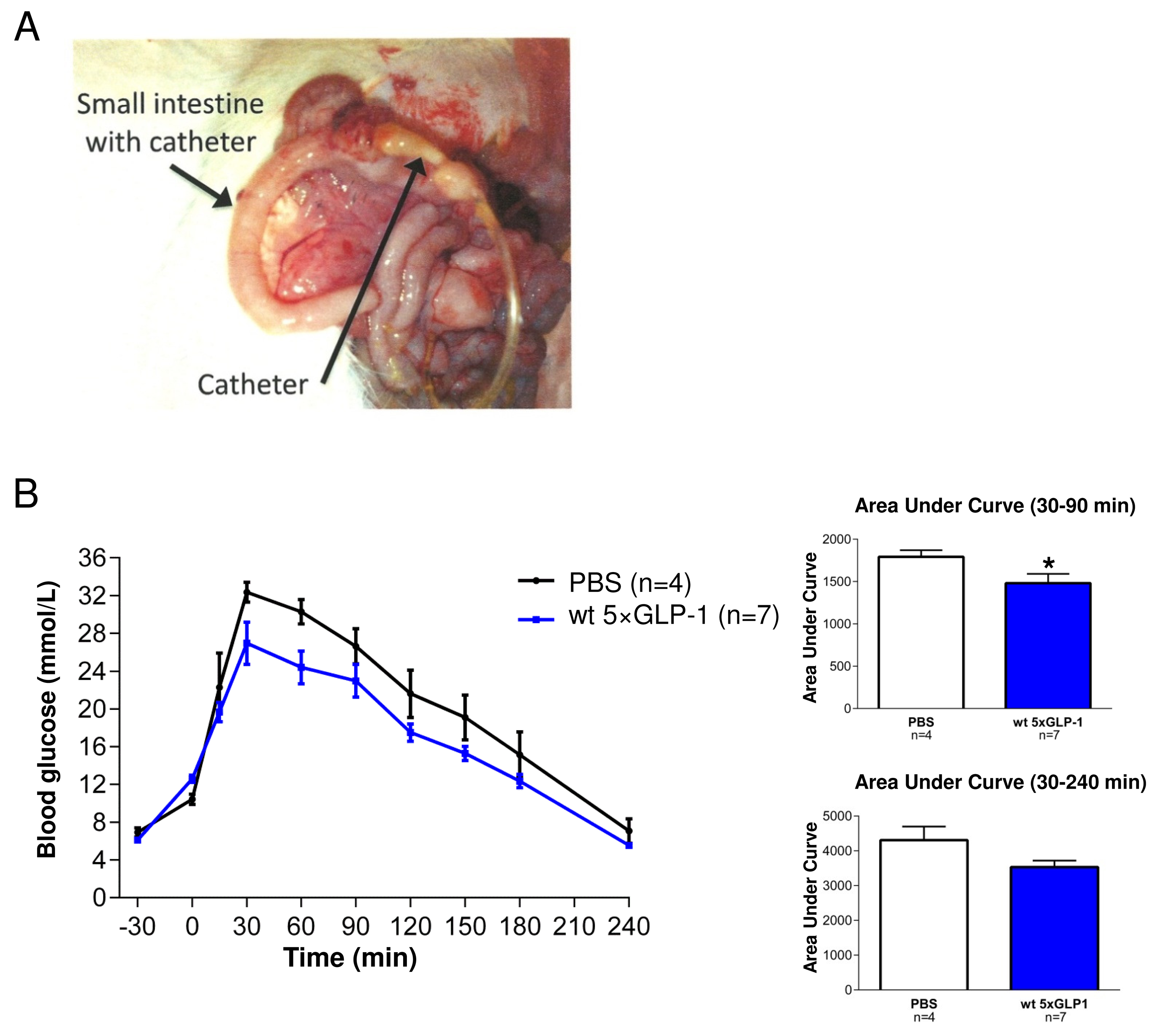

Supplement: S1 Fig — (A) The small intestine of GK rats was cannulated with a catheter to facilitate administration of the 5×GLP-1 peptide intra-intestinally. (B) Wt 5×GLP-1 was administered intra-intestinally at 5 mg/kg body weight to GK rats 30 min prior to the glucose challenge (glucose: 2 g/kg of body weight). The area under the curve (AUC) for glucose levels for the period corresponding to 30 to 240 min is shown. Data are presented as the mean ± SEM (PBS group, n = 4; 5×GLP-1 group, n = 7). *p<0.05 versus PBS, unpaired t-test with Welch’s correction. (TIF) [file pone.0162733.s001.tif]

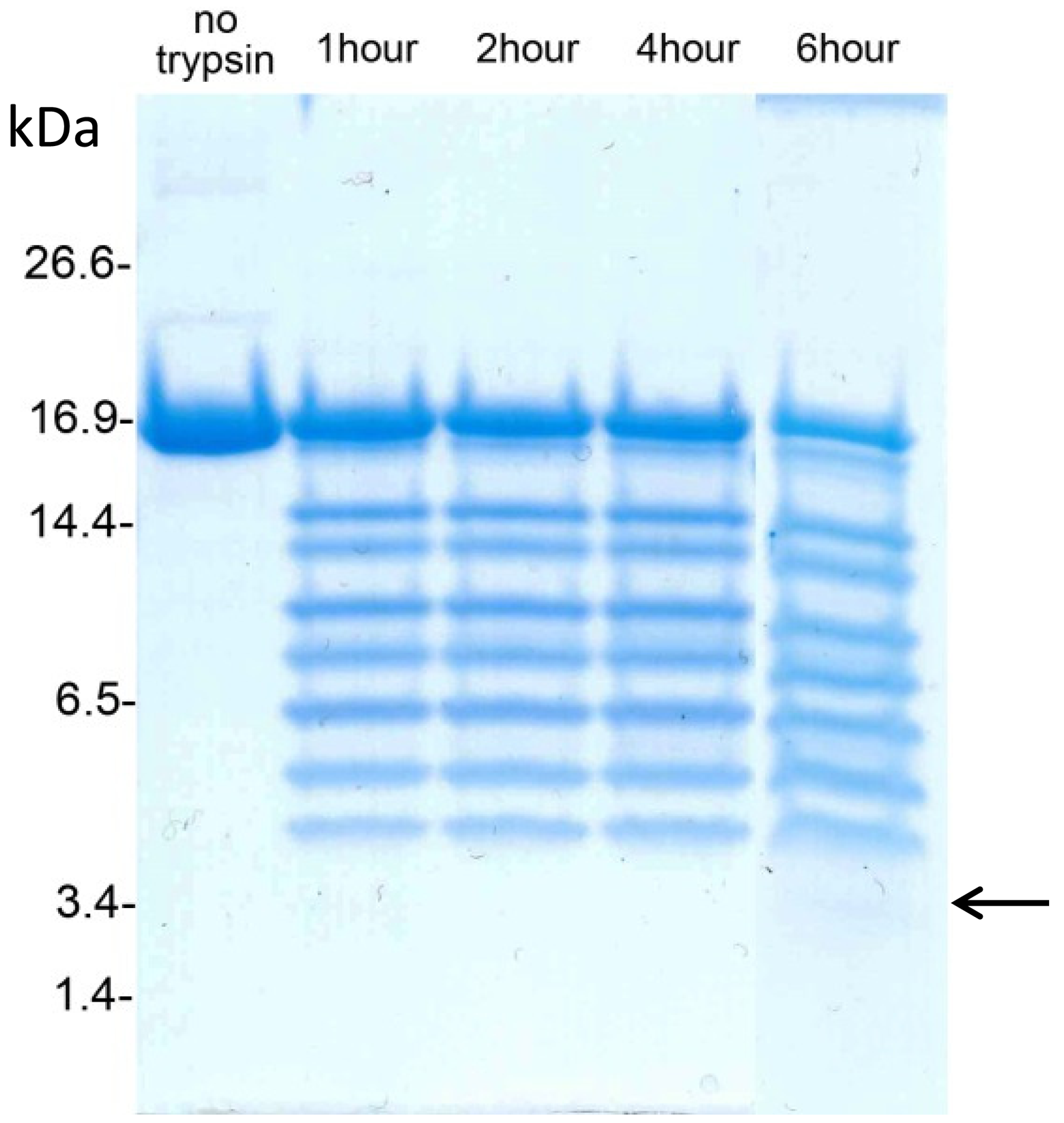

Supplement: S2 Fig — The wt 5×GLP-1 purified from E. coli was digested using a trypsin spin column for 1, 2, 4 or 6 hours. The purified protein (2.5 μg) and digested protein (2.5 μg) were loaded in parallel on a SDS-PAGE gel and detected by Coomassie stain. The arrow indicates the size of GLP-1 monomer (3.4kDa). (TIF) [file pone.0162733.s002.tif]

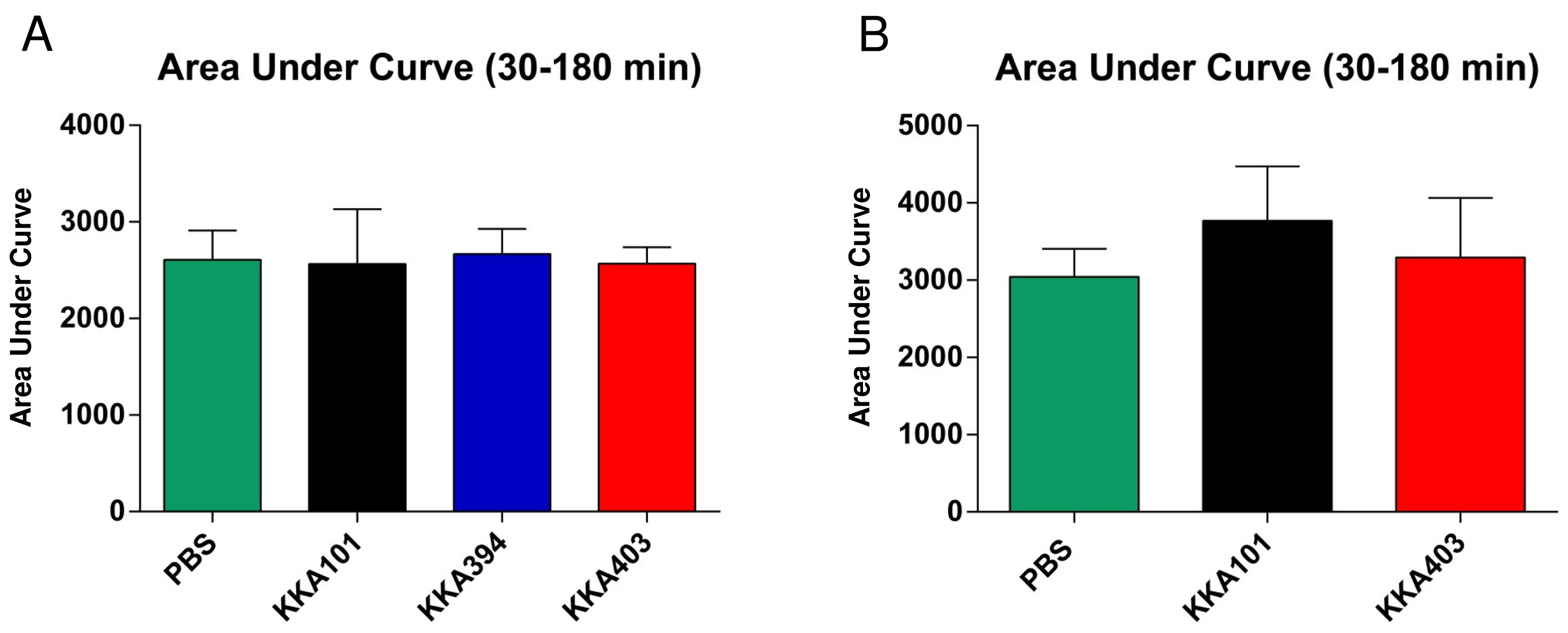

Supplement: S3 Fig — (A) The area under the curve (AUC) for glucose levels for the period corresponding to 30–180 min after the 7-day feeding experiment (n = 6). (B) The AUC for glucose levels for the period corresponding to 30–180 min after the 14-day feeding experiment (n = 7). Data are presented as the mean ± SEM. (TIF) [file pone.0162733.s003.tif]
