# Supplementary material for: Oral Delivery of Pentameric Glucagon-Like Peptide-1 by Recombinant Lactobacillus in Diabetic Rats
Source: PLoS One. 2016 Sep 9;11(9):e0162733. doi: 10.1371/journal.pone.0162733 (PMC5017604; doi:10.1371/journal.pone.0162733)
Supplement: S1 Table — (DOCX) [file pone.0162733.s004.docx]

**Table S1: Synthetic genes used for plasmid construction**

| **Gene name** | **Sequence** |
| --- | --- |
| wt 5xGLP-1 | CCATGGATCGTCATGGTGAAGGCACCTTTACGTCAGATGTTTCAAGCTATCTGGAAGGCCAAGCTGCTAAGGAATTTATAGCTTGGTTAGTTAAGGGCCGTCACGGTGAAGGCACGTTTACAAGTGATGTCTCAAGTTATCTAGAGGGTCAGGCGGCAAAGGAATTTATCGCTTGGTTAGTCAAAGGACGACATGGTGAAGGTACTTTCACTTCGGATGTATCTAGCTACTTAGAAGGGCAGGCCGCAAAAGAATTTATTGCCTGGCTTGTGAAAGGCAGACACGGTGAGGGAACCTTTACTAGTGACGTTTCCAGTTATCTCGAAGGACAAGCAGCTAAAGAGTTTATTGCGTGGCTAGTTAAGGGACGCCACGGAGAAGGCACATTCACCTCGGACGTTAGCAGTTATCTTGAAGGTCAAGCAGCGAAAGAGTTTATTGCATGGCTTGTCAAAGGGCGTGCGGCCGC |
| trp 5xGLP-1 | CCATGGGCCGACATGGAGAAGGTACTTTCACGAGTGATGTTTCTTCATATTTGGAAGGGCAAGCAGCTCAAGAATTTATTGCCTGGCTTGTAGATGGAAGACATGGCGAAGGCACATTTACGAGTGATGTCAGCTCATATCTTGAAGGCCAAGCTGCACAAGAATTTATCGCTTGGTTGGTAGATGGCCGCCACGGCGAAGGCACCTTTACATCGGATGTCTCCTCATACTTGGAAGGCCAAGCAGCGCAAGAATTTATTGCTTGGCTTGTTGATGGCCGTCATGGAGAAGGCACTTTTACAAGTGATGTTTCAAGCTATTTGGAAGGACAAGCTGCTCAGGAATTTATTGCGTGGCTTGTCGATGGTCGTCATGGCGAAGGTACATTCACGAGCGATGTCAGTTCATATTTGGAGGGTCAGGCGGCCCAGGAATTTATTGCATGGTTGGTTGATGGTCGAGCGGCCGC |
