# Supplementary material for: Oral Delivery of Pentameric Glucagon-Like Peptide-1 by Recombinant Lactobacillus in Diabetic Rats
Source: PLoS One. 2016 Sep 9;11(9):e0162733. doi: 10.1371/journal.pone.0162733 (PMC5017604; doi:10.1371/journal.pone.0162733)
Supplement: S2 Table — (DOCX) [file pone.0162733.s005.docx]

**Table S2 Effects of GLP-1 peptides on insulin release from isolated rat pancreatic islets**

| **Addition to incubation medium** | | | **Insulin release (μU/islet/h)** | | |
| --- | --- | --- | --- | --- | --- |
| **Glucose (mM)** | **Peptide (nM)** | **GLP-1** | | **GLP-1-Gly8** | |
| 16.7 | none | | 61.3 ± 8.7 | | 129 ± 14 |
|  | 1 | | 88.0 ± 17.5 | | 143 ± 8 |
|  | 10 | | 119 ± 25 | | 183 ± 17 |
|  | 100 | | 153 ± 34 | | 168 ± 4 |
