# Supplementary material for: Oral Delivery of Pentameric Glucagon-Like Peptide-1 by Recombinant Lactobacillus in Diabetic Rats
Source: PLoS One. 2016 Sep 9;11(9):e0162733. doi: 10.1371/journal.pone.0162733 (PMC5017604; doi:10.1371/journal.pone.0162733)
Supplement: S3 Table — (DOCX) [file pone.0162733.s006.docx]

**Table S3: Signal peptides tested for secretion of GLP-1 peptide with right N-terminal cleavage**

| **Signal peptide** | **Peptide sequence** | **Secretion** | **Right cleavage** |
| --- | --- | --- | --- |
| **SP1-NZ9000** | MANLKKKLTLTGLMTAGLLLLSGCVQT | Yes | No |
| **SP2-NZ9000** | MAKIVLVALAAMAFIMGGVINA | Yes | No |
| **SP3-Il1403_1** | MAKIVKNLQVIVALVFALLMLSACFTSKSELPRNTSNA | Yes | No |
| **SP4-NZ9000** | MAKLLTGLLVTAGLLSFSLFIKTDSVSA | Yes | No |
| **SP5-M6** | MAKNNTNRHYSLRKLKKGTASVAVALSVIGAGLVVNTNEVSA | Yes | No |
| **SP6-lp_3050** | MAKFNFKTMLLLVLASCVFGVVVNVTTSLGPQTAITAQA | Yes | No |
| **SP7-lp_2578** | MARKLVGYMLSMLTVILALFMLGSTAHA | Yes | No |
| **SP-LP_374** | MVKLRQVLKKILIGLMVFVLVFTAFSSSVDTVSA | Yes | No |
